# A Phase IIb Randomized Controlled Trial Investigating the Effects of Tocotrienol-Rich Vitamin E on Diabetic Kidney Disease

**DOI:** 10.3390/nu13010258

**Published:** 2021-01-18

**Authors:** Yan Yi Koay, Gerald Chen Jie Tan, Sonia Chew Wen Phang, J-Ian Ho, Pei Fen Chuar, Loon Shin Ho, Badariah Ahmad, Khalid Abdul Kadir

**Affiliations:** Jeffrey Cheah School of Medicine and Health Sciences, Monash University Malaysia, Bandar Sunway, Selangor 47500, Malaysia; gctan73@gmail.com (G.C.J.T.); sonia.phang1@monash.edu (S.C.W.P.); ian.ho1996@gmail.com (J.-I.H.); peifen0307@gmail.com (P.F.C.); ho.loon.shin@monash.edu (L.S.H.); khalid.kadir@monash.edu (K.A.K.)

**Keywords:** tocotrienols, vitamin E, diabetic kidney disease, antifibrotic, antioxidant, anti-inflammatory, transforming growth factor-beta 1 (TGF-β1)

## Abstract

Diabetic kidney disease (DKD) is a debilitating complication of diabetes, which develops in 40% of the diabetic population and is responsible for up to 50% of end-stage renal disease (ESRD). Tocotrienols have shown to be a potent antioxidant, anti-inflammatory, and antifibrotic agent in animal and clinical studies. This study evaluated the effects of 400 mg tocotrienol-rich vitamin E supplementation daily on 59 DKD patients over a 12-month period. Patients with stage 3 chronic kidney disease (CKD) or positive urine microalbuminuria (urine to albumin creatinine ratio; UACR > 20–200 mg/mmol) were recruited into a randomized, double-blind, placebo-controlled trial. Patients were randomized into either intervention group (*n* = 31) which received tocotrienol-rich vitamin E (Tocovid SupraBio^TM^; Hovid Berhad, Ipoh, Malaysia) 400 mg daily or a placebo group which received placebo capsules (*n* = 28) for 12 months. HbA1c, renal parameters (i.e., serum creatinine, eGFR, and UACR), and serum biomarkers were collected at intervals of two months. Tocovid supplementation significantly reduced serum creatinine levels (MD: −4.28 ± 14.92 vs. 9.18 ± 24.96), *p* = 0.029, and significantly improved eGFR (MD: 1.90 ± 5.76 vs. −3.29 ± 9.24), *p* = 0.011 after eight months. Subgroup analysis of 37 patients with stage 3 CKD demonstrated persistent renoprotective effects over 12 months; Tocovid improved eGFR (MD: 4.83 ± 6.78 vs. −1.45 ± 9.18), *p* = 0.022 and serum creatinine (MD: −7.85(20.75) vs. 0.84(26.03), *p* = 0.042) but not UACR. After six months post washout, there was no improvement in serum creatinine and eGFR. There were no significant changes in the serum biomarkers, TGF-β1 and VEGF-A. Our findings verified the results from the pilot phase study where tocotrienol-rich vitamin E supplementation at two and three months improved kidney function as assessed by serum creatinine and eGFR but not UACR.

## 1. Introduction

Diabetic kidney disease (DKD) is a debilitating microvascular complication of diabetes progressing to end-stage renal failure (ESRF). To date, the only management available for ESRF is restricted to hemodialysis and renal replacement therapy [1]. Hence, early diagnosis and adequate management are crucial to halt the progression of DKD to ESRF.

The pathophysiology of DKD is complex, multifactorial, and is not fully elucidated. As a result of chronic hyperglycemia, four major metabolic pathways such as protein kinase C, the polyol pathway, advanced glycation end products (AGEs), and the hexosamine pathway are activated. The overproduction of these end-products and by-products of these pathways results in microvascular and macrovascular complications of diabetes [2]. Specifically, NFκB activation and PKC activation lead to abnormal upregulation of vascular and endothelial activity in renal glomeruli in diabetes [3].

Vitamin E tocopherols and tocotrienols have potent antioxidant and anti-inflammatory properties [4]. Both classes of compounds are subdivided into four isomers, alpha, beta, gamma, and delta-tocotrienols (*α*, *β*, *γ*, *δ*), that are naturally found in a number of vegetable oils, wheat germ, barley, and certain types of nuts and grains [4]. Tocotrienols have gained scientific interest after recent studies have demonstrated its superiority over tocopherols. The unique ability of tocotrienols to distribute uniformly in the cellular bilayer and readily penetrate into tissues with saturated fatty layers made it 50 times more potent as an antioxidant compared to tocopherols [5,6,7]. The role of vitamin E supplementation for metabolic diseases and diabetes-associated complications remains controversial. [3] The MICRO-HOPE study (2000) showed no significant renoprotective and cardioprotective outcomes with daily administration of tocopherol for 4.5 years [8]. A few systematic reviews concluded there was insufficient evidence to support the role of vitamin E as tocopherols for supplementation; there was no significant improvement in diabetic control as assessed by HbA1c levels and fasting sugar levels in the unselected population [9,10,11]. However, most of these studies pertain to tocopherol and not tocotrienol; studies that were exclusively on tocotrienol and diabetes only consisted of approximately 3–4% of all research studies on vitamin E [5].

Current perspectives regarding the mechanism of action of tocotrienol on the kidneys were mainly on oxidative and inflammatory stress, but this may not reflect the whole picture. This was supported by our pilot study; while there was a significant improvement in renal function after 3 months of supplementation, no correlation was found between renal function and oxidative stress or inflammatory stress markers. Furthermore, this renal improvement was not related to changes in HbA1c, blood pressure, or BMI [12,13].

There is growing evidence that transforming growth factor beta 1 (TGF-β1), a fibrogenic cytokine, is crucial in mediating the cascade of events leading to DKD. However, its modulation by tocotrienol in DKD remains unexplored [14]. Siddiqui et al. (2013) reported that tocotrienol-rich fraction supplementation improved renal function in T2DM rats via suppression of TGF-β1, collagen type IV, and fibronectin in the kidney [15]. Cojocel et al. (2015) showed that vitamin E modulated TGF-β1 mRNA overexpression, hence it may be effective in early stages of DKD [16]. TGF-β1 may be a possible molecular marker modulated by tocotrienols and could play a role in the pathogenesis of DKD.

The primary aim of this study was to determine the effect of high-dose tocotrienol-rich vitamin E on DKD, as assessed by serum creatinine and estimated glomerular filtration rate (eGFR) and urine to albumin ratio (UACR). The secondary aim was to investigate the effects of tocotrienol-rich vitamin E on antifibrotic biomarkers, namely TGF-β1.

## 2. Materials and Methods

### 2.1. Study Design and Participants

This is a multicenter, placebo-controlled, double-blind, prospective randomized controlled trial to evaluate the effects of tocotrienol-rich vitamin E on diabetic kidney disease patients. This trial commenced in 2019 and was conducted at two clinical research centers in Malaysia, namely the Clinical Research Centres (CRC) of Monash University Sunway and at Monash University Clinical School Johor Bahru (CSJB). The patients were followed up closely at intervals of two months over a year until March 2020 followed by a six-month washout period until September 2020.

Study candidates were recruited from a pool of diabetic patients who are reviewed on a regular basis. Potential patients with renal complications were notified to attend for possible inclusion into the study, where all study-related procedures and blood investigations were taken and sent to a designated national certified pathology laboratory. Prior to every visit, patients were reminded to fast for at least 8 h, omit their morning dose of antidiabetic medications, and avoid strenuous exercise the night before. Premenopausal patients with ongoing menses or those who had fallen ill during the scheduled visits were permitted to reschedule their appointment dates.

### 2.2. Inclusion and Exclusion Criteria

The inclusion criteria for patients included an age range of 18 to 75 years old with stable glycemic control assessed by HbA1c between 6 and 9% (not more than 10% fluctuation over the last 2 months). Resting blood pressure must be less than 150/90 mmHg. Eligible patients must have chronic kidney disease secondary to T2DM defined by either a reduced eGFR (30–60 mL/min/1.73 m^2^) or microalbuminuria, UACR > 20–200 mg/mmol. This criterion of stage 3 CKD is outlined by the KDOQI guidelines. Patients with stage 5 CKD (eGFR < 15 mL/min/1.73 m^2^) were excluded due to the lack of information on drug safety issues.

Patients with non-diabetic kidney disease such as kidney stones, minimal change disease, IgA nephropathy, idiopathic membranous nephropathy, acute tubulointerstitial nephritis, and untreated urinary tract infection were excluded. The exclusion criteria include unstable or severe chronic illness (active cancer, acute coronary syndrome, inflammatory disorders, and liver disease), unstable eye disease, or pregnancy. Patients who had consumed any form of water-soluble supplements such as vitamin B, C, and glutathione, or fat-soluble antioxidants such as vitamin A, D, E, and K during the past month were told to return after one month of washout.

### 2.3. Ethics

The study was carried out in accordance with the Declaration of Helsinki and the study protocol was approved by Monash University Human Research Ethics Committee (MUHREC; Clayton, Australia) (Project number: 12090). This trial was registered in the Australian New Zealand Clinical Trials Registry (ANZCTR; Camperdown, Australia), with the trial registration number ACTRN12619001568101.

### 2.4. Screening Visit

Written informed consent was obtained from all patients before any procedures were performed during the visit. A thorough history-taking and physical examination were performed, followed by anthropometric measurements (i.e., waist circumference, weight, and height). Blood pressure and pulse were measured thrice to obtain an average reading. Blood and urine samples were collected for HbA1c, fasting blood glucose, renal profile, lipid profile, liver function tests, and full blood count. Urine analysis by dipstick test, urine pregnancy test, and UACR were conducted as well. An electrocardiogram (ECG) was also taken to ensure subject eligibility. Patients that fulfilled the criteria were notified and invited to return for randomization two to four weeks after screening.

### 2.5. Randomization and Blinding

Patients were randomized into either the intervention or control group in a 1:1 ratio by an independent party using a computer-generated random sequence. Stratification was performed according to gender, duration of diabetes, and HbA1c levels. Patients in the intervention group were given 200 mg tocotrienol-rich vitamin E (Tocovid SupraBio^TM^; Hovid Berhad, Ipoh, Malaysia) twice daily, while the control group were given identical looking capsules (tocotrienol-free palm oil capsules) twice daily. Tocovid SupraBio^TM^ and the placebo capsules were sponsored by ExcelVite, Malaysia and manufactured by Hovid Pharmaceuticals Berhad, Malaysia.

To prevent selection or performance bias, the identity of the investigational products was kept confidential by the manufacturers from the researchers and patients until the end of the study. An independent party was responsible to assign drug codes for all patients to conceal the allocation from both researchers and patients.

### 2.6. Sample Size

The power calculations were based on the ability to detect a 30% reduction in UACR in the primary analysis of tocotrienol-rich vitamin E (Tocovid SupraBio^TM^) compared to placebo, assuming a 5% standard deviation (SD) of effect (α = 0.05 and 1 − β = 0.8) and an anticipated dropout rate of 10%. To fulfill these specifications, a total of 58 patients were needed.

### 2.7. Follow-Up Visits

Patients were followed up at two-month intervals over the course of a year to monitor for adverse drug events and compliance to treatment. During each follow up visit, patients returned their drug bottles for compliance assessment via pill counting. Blood pressure, anthropometric measurements, finger-prick fasting glucose test, and urine dipstick were routinely performed during each monitoring visit. Fasting blood samples in the fasting state were collected during every visit for renal profile (serum creatinine, estimated glomerular filtration rate, eGFR, urine to albumin creatinine ratio, UACR), safety tests (lipid profile and liver profile), measurement of vitamin E levels, and biomarkers. ECG was performed as part of the safety tests. Patients were advised to discontinue participating in the study if they experienced serious adverse events or became pregnant during the course of the study.

As per local guidelines, patients in both groups received the standard of care and were required to be reviewed by their regular physician or endocrinologist, alongside the routine review by a research doctor during the follow-up visits. Any changes in medication during the study were documented. The identity of the study drugs was revealed by ExcelVite at the end of study. Drug A was identified as tocotrienol-rich vitamin E (Tocovid SupraBio^TM^) while drug B was the placebo.

### 2.8. Assessment of Outcomes

The primary outcome variables of this study were eGFR, serum creatinine, and UACR. The secondary outcome variables were HbA1c, urea, serum uric acid, TGF-β1, and VEGF-A.

### 2.9. Serum Creatinine, eGFR, Liver Function Test and Lipid Profile, HbA1c

During the screening visit and every follow up visit, 18mL of fasting blood samples were collected from each patient. These blood samples were collected in serum-separating tubes (SST) and were centrifuged (Eppendorf Centrifuge 5702R, Hamburg, Germany) at 3600 rpm for 15 min to obtain serum. Sera was transferred into 1mL Eppendorf tubes. A portion of these serum samples were then sent to a national certified pathology lab for analysis of serum creatinine, eGFR, liver function, and lipid profile (ARCHITECT, Abbott diagnostic, Abbott Park, IL, USA) on the same day. The coefficient variances for these tests were below 6%. eGFR was calculated using the CKD-EPI formula and presented as milliliters per minute per 1.73 m^2^. HbA1c analysis was performed with a Cobas Integra 400 plus analyzer, (Roche Diagnostics, Quebec, Canada) with a measuring range of 4.3–18.8% with coefficient variances (CV) of less than 5%.

### 2.10. TGF- β1, VEGF-A, Tocotrienol, Tocopherol Levels

The remaining serum samples kept in the Eppendorf tubes were stored in a −80 °C fridge on the same day. To minimize inter-assay variation, serum biomarker analysis was performed at the end of the study, on a batch-to-batch basis. These biomarkers were measured using enzyme-linked immunosorbent assay (ELISA) in duplicates and quantified by calorimetric method. An ELISA plate reader (Eon Biotek Instruments, Winooski, VT, USA) was used. The ELISA kits include Elabscience E-EL-0162 (Houston, TX, USA) for serum TGF-β1 and Elabscience E-EL-H0111 (Houston, TX, USA) for serum VEGF. These kits had intra-assay coefficient variances of 4% and inter-assay coefficient variances of 8%. Blood samples collected in EDTA tubes were centrifuged at 3600 rpm for 15 min to obtain plasma samples. Plasma tocotrienols and tocopherol were measured for all patients using high-performance liquid chromatography (HPLC; HPLC 1200, Agilent, Santa Clara, CA, USA) with a fluorescence detector (FLD) based on a method by Che et al. [17] with modification.

### 2.11. Urine Albumin Creatinine Ratio UACR

Collected urine samples were sent to the same pathology lab on the same day to assess for UACR. The UACR kit (Abbott diagnostic ARCHITECT, Abbott Park, IL, USA) has a coefficient variance of less than 5% for urine creatinine and less than 6% for microalbuminuria. To obtain an average reading of UACR at baseline, two UACR readings were taken on two different visits (screening and visit 1).

### 2.12. Statistical Analysis

All statistical analysis was performed using SPSS^®^ Statistics, Version 26.0, Illinois (USA), IBM; 2016. Two-sided *p* values were calculated; the probability value of ≤0.05 was used to reject the null hypothesis.

Baseline measurements such as sociodemographics, status of diabetes, and renal parameters between two groups were assessed using an independent *t*-test for continuous variables, while a Chi-square test was performed for categorical variables.

The mean differences of each parameter were calculated between follow-up visits and baseline visit and recorded as continuous variables. Subsequently, these variables were then evaluated for normality using the Shapiro–Wilk test. Parametric continuous variables were evaluated using an independent t-test, while the Mann–Whitney test was used for non-parametric continuous variables. Descriptive statistics of normally distributed data were reported as mean and standard deviations, and median (interquartile) for non-normally distributed data. Categorical data were presented as percentages. The effect size of the study was analyzed using Cohen’s d method and calculated based on the independent *t*-test. The value of d signifies the magnitude of effect size; values > 0.8 depict a “large” effect size which simply means the results are influential.

To depict real-life situations, a modified intention-to-treat analysis approach was used in this trial. Missing secondary data lost to follow-up were imputed based on the last observation carried forward method, while missing values at random were imputed using the series mean function in SPSS. This form of imputation is valid, as the proportion of missing data constitutes less than 10% of the total sample size.

## 3. Results

A total of 160 patients with T2DM were screened over a period of 2 months. Of these, 60 patients met the inclusion criteria and were invited to participate in the clinical trial. Fifty-nine patients turned up for randomization—31 in the intervention group and 28 in the placebo group.

At the end of the trial, 9 out of the 59 patients did not return for follow up, 5 of which were unable to attend the last follow up visit due to the COVID-19 pandemic, 3 withdrawn due to adverse events unrelated intervention, and 1 moved/migrated from the study area. However, all 59 patients were retained and analyzed per modified intention-to-treat analysis.

**Figure 1:**
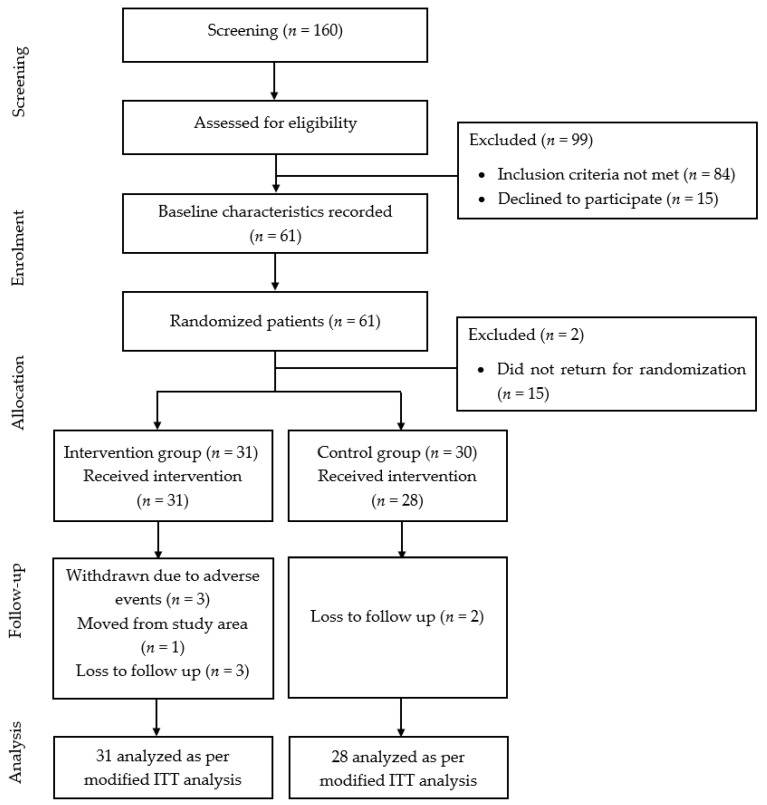
Summary of patient flow diagram. *n*: number of patients.

A summary of the patient flow diagram is shown in Figure 1.

### 3.1. Baseline Characteristics

The baseline characteristics of 59 patients are shown in Table 1. The median age of the patients was 67, with male as the dominant gender (64.4%). The cohort was predominantly made up of the Malay ethnicity, representative of the national demographics of Malaysia. Most of the patients have stage 3A CKD with moderately increased UACR, and the average eGFR was 55 mL/min/1.73 m^2^. The average systolic blood pressure (SBP) and diastolic blood pressure (DBP) readings were 132/76 mmHg, respectively. Based on the Asian criteria BMI by WHO, the average BMI of the patients was 28.54 kg/m^2^ which is classified as obese [18].

### 3.2. Six Months Post-Tocotrienol-Rich Vitamin E Supplementation

Table 2 presents a comparison of the treatment changes between the intervention group and placebo at the end of six months. An increment in eGFR denotes a positive change, whereas a reduction in serum creatinine and UACR signifies renal improvement.

At the end of six months, tocotrienol-rich vitamin E supplementation significantly reduced serum creatinine (mean difference, MD = −13.3) and increased eGFR (MD = 6.0 mL/min/1.73 m^2^) compared to the placebo. Although UACR levels in the control group were much reduced compared to the intervention group, this change was not significant. There were no significant changes seen in the serum biomarker TGF-β1 nor in VEGF-A. No significant changes were seen in HbA1c levels, blood pressure, urea, and uric acid.

### 3.3. Twelve Months Post-Tocotrienol-Rich Vitamin E Supplementation

Based on Table 3, tocotrienol-rich vitamin E supplementation significantly improved renal function at the end of eight months. There was a significant increase in eGFR (MD = 5.1 mL/min/1.73 m^2^) and reduction in serum creatinine (MD = 13.4 umol/L) compared to the placebo. However, at twelve months supplementation, there were no changes in these renal parameters. This result suggests that the renoprotective effects of tocotrienol-rich vitamin E (Tocovid SupraBio^TM^) were only sustained until eight months post supplementation. There was a significant change in urea at twelve months of supplementation when compared to the placebo. There were no significant changes in other parameters, nor in TGF-β1 nor in VEGF-A, which are important markers that mediate DKD when comparing both groups.

### 3.4. Correlation of Renal Parameters with TGF-β1 and VEGF-A

Table 4 showed that there was no significant correlation between TGF-β1 with serum creatinine and eGFR. Furthermore, there was also no correlation found between VEGF-A with serum creatinine and eGFR.

### 3.5. Subgroup Analysis

Patients with a glomerular filtration rate of 30–60 mL/min/1.73 m^2^ and stage 3 CKD at baseline were stratified into a subgroup. Based on these selection criteria, 20 patients were in the treatment group and 17 in the placebo group.

Table 5 showed a sustained increase in creatinine clearance after 6 months of tocotrienol-rich vitamin E supplementation (*p* = 0.05 *) in this subgroup. There was a 7.23 umol/L decrease in serum creatinine levels in the intervention group, while there was an increment of 5.82 umol/L in the placebo. Moreover, there were no significant changes in blood pressure and HbA1c.

Based on Table 6, tocotrienol-rich vitamin E supplementation significantly increased creatinine clearance and eGFR at twelve months of supplementation. These changes were sustained throughout the study period of a year. There was a reduction in serum creatinine levels of 7.85 umol/L in the intervention group with a corresponding 4.83 mL/min/1.73 m^2^ increase in eGFR in the intervention group.

**Figure 2:**
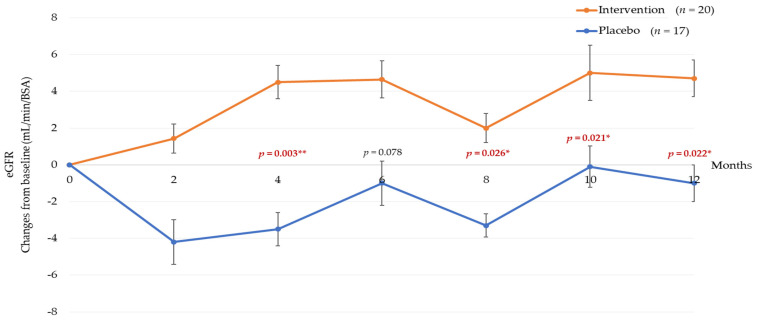
Graph of overall trend of estimated glomerular filtration rate (eGFR) for 12 months. Data are presented as mean ± standard deviation. * Significant at *p* < 0.05, ** *p* < 0.001.

Figure 2 illustrates the trend of the eGFR of 37 patients from the subgroup analysis. A downtrend in eGFR levels is observed in the placebo group, whereas an uptrend is seen in the intervention group. On average, patients in the placebo group have a rate of eGFR declined in approximation to 2.26 mL/min/1.73 m^2^ over a year which corresponds to the natural history and progression of DKD. More often, the status of eGFR will be used to assess the progression of DKD. Patients with declined eGFR of ≥2 mL/min/1.73 m^2^ per year will be identified as progressors and have higher chances to eventually progress to ESRF [19].

### 3.6. Post Washout Analysis

The post washout section of the clinical trial protocol was disrupted by the nationwide implementation of the movement control order (MCO) as a public health measure to contain the COVID-19 pandemic. The MCO was executed from March until September 2020. During this time, patients were kept in contact via telephone to assess how they were doing. At 6 months post washout, which coincided with the temporary lifting of the MCO, patients were called to return to the clinic, where they were reviewed and blood and urine samples were collected for post washout analysis. The table below shows the comparison of renal parameters at 12 months Tocovid supplementation and at 6 months post washout between intervention and placebo groups.

Post washout, the intervention group showed a statistically significant reduction in UACR by 19.3 mg/mmol in contrast to the placebo group, which showed an increase in UACR by 4.6 mg/mmol. However, there were no significant differences in terms of HbA1c, serum creatinine, eGFR, and uric acid between the groups.

## 4. Discussion

Diabetic kidney disease is clinically diagnosed with the presence of persistent high urine albumin-to-creatinine ratio of ≥30 mg/g and/or sustained declined in eGFR below 60 mL/min/1.73 m^2^ [20]. Classically, the presentation of DKD progresses from the appearance of urinary microalbuminuria, followed by an increase in macroalbuminuria and eventually declined in renal function [21]. However, recent evidence has shown that approximately 40% of those that developed renal functional impairment never developed albuminuria [20]. Anyanwagu et al. (2019) found a higher mortality risk associated with the non-albuminuric phenotype compared to individuals with albuminuria but normal eGFR [22]. This finding was supported by the FIELD study which showed that the non-albuminuric phenotype was associated with a higher risk of all-cause mortality [23]. As a result, this necessitates for an effective treatment to delay the progression of DKD and improve the quality of life of these patients.

**Figure 3:**
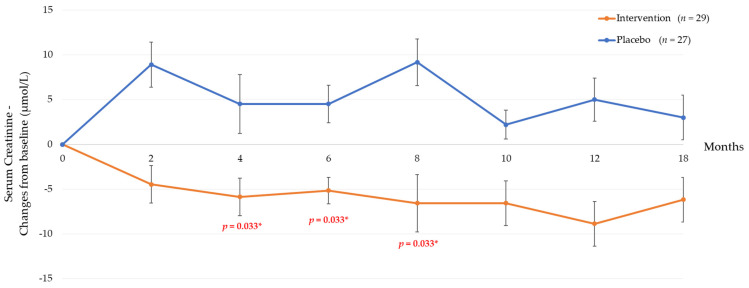
Changes in serum creatinine in intervention and placebo group throughout the study period of 18 months. Data are presented as mean ± standard deviation. * Significant at *p* < 0.05.

This clinical trial demonstrated that oral supplementation of tocotrienol-rich vitamin E significantly improved renal function as assessed by serum creatinine and eGFR but not UACR (Table 2 and Table 3, Figure 3). However, these effects were observed only up until eight months. In addition, the findings of this study are consistent with our pilot phase study, demonstrating that patients who were supplemented with tocotrienol-rich vitamin E at 3 months had a significant increase in eGFR and creatinine clearance but no significant effect on urine protein excretion.

Furthermore, tocotrienol-rich vitamin E supplementation may be more beneficial for DKD patients with an eGFR of 30–60 mL/min/1.73 m^2^ as they persistently benefited from tocotrienol-rich vitamin E supplementation throughout the 12 months study period although there was no improvement in UACR (Table 5 and Table 6).

Although, tocotrienol-rich vitamin E significantly increased eGFR and creatinine clearance, the exact mechanism which leads to these functional changes in the kidney remains unknown. Potentially, this change could be due to chronic antifibrotic effects. Vitamin E concentrations in the renal cortex were associated with downregulation of TGF-β1 levels and reduction in glomerular expansion [24,25,26]. Pathological matrix collection, fibrotic contraction and replacement of structural function lead to the demise of organ function. As fibrosis progresses, sclerotic changes take place, promoting loss of glomerular filtration barrier, autoregulation of peritubular capillaries, and ultimately, a decline in glomerular filtration [27,28].

Furthermore, it is unlikely that the changes in eGFR are secondary to drug-induced hyperfiltration [29]. Interventions that lead to hyperfiltration would be expected to lead to a rapid deterioration in renal function [29]. In contrast, when DKD patients with stage 3 CKD (eGFR between 30 to 60 mL/min/1.73 m^2^) were supplemented with tocotrienol-rich vitamin E, they appeared to benefit from a sustained increase in eGFR over one year.

Our earlier pilot phase study had reported a sustain improvement in creatinine clearance even 6 to 9 months after the intervention had been withdrawn [13]. Furthermore, there were no serious adverse events that were related to the kidney such as end-stage renal failure or clinically meaningful drastic loss of eGFR. Therefore, there was no substantiated evidence suggesting the increase in eGFR was associated with pressure-mediated hyperfiltration. In contrast to the previous pilot phase, in this study, cessation of Tocovid supplementation for 6 months did not improve serum creatinine and eGFR; however, there was a significant reduction in UACR as compared to the placebo group (Table 7, Figure 3). Nevertheless, long-term observation is warranted to elucidate the true biological significance of increased eGFR.

Our study demonstrated no significant changes in serum TGF-β1 levels with tocotrienol-rich vitamin E supplementation (Table 4). However, the lack of significant effects may be due to the limitation of TGF-β1 as a biomarker for DKD. Although, meta-analysis by Xin et al. (2016) showed a positive association between serum TGF-β1 levels with DKD, it is not without the shortcoming of small sample size (246 DKD patients) and high heterogeneity due to the lack of discrete DKD diagnostic criteria [30]. Similarly, this study did not show any significant changes in HbA1c, VEGF-A, or NGF. In our earlier pilot study using similar doses of tocotrienol-rich vitamin E, there were no significant changes in the serum biomarkers AGE, RAGE, Nε-CML, Cystatin C, TNFR-1, MDA, and thromboxane, although significant improvement in renal function was detected.

Nevertheless, tocotrienol-rich vitamin E significantly reduced serum creatinine and improved eGFR compared to the placebo, indicating there are different pathways or molecular mediators that were modulated by tocotrienol-rich vitamin E that are yet to be discovered. One potential limitation of the study is the sample size. Based on the power calculations performed, the sample size of 59 patients was sufficient to detect statistically significant changes. Novel biomarkers of diabetic kidney disease should be explored further. Future studies could apply proteomics to study protein expression profiling and targeted protein quantification. This may bridge the knowledge gap to exploring other possible molecular mechanisms that are modulated by tocotrienol-rich vitamin E [31]. Further research is warranted to elucidate the complex nature of tocotrienol-rich vitamin E and its effects on DKD.

In conclusion, this is the first clinical trial that demonstrated that tocotrienol-rich vitamin E supplementation for 12 months was able to ameliorate the progression of DKD, especially in patients with stage 3 CKD (eGFR 30–60 mL/min/1.73 m^2^). Tocotrienol-rich vitamin E supplementation improved renal function, as assessed by a significant reduction in serum creatinine and significant increment in eGFR when compared to placebo, but not urine albumin excretion. At 6 months post washout, there was no improvement in serum creatinine and eGFR. Given the complexity of tocotrienol and the pathophysiology of diabetic kidney disease, the pathway in which tocotrienols exert on the renal function remains elusive. Further studies are warranted to determine the mechanism of action of tocotrienols.

## Figures and Tables

**Table 1:** Baseline characteristics of 59 patients before intervention.

Baseline Characteristic	Tocovid (*n* = 31)	Placebo (*n* = 28)	*p*-Value	Total (*n* = 59)
Age (years)	66 (13)	70 (13)	0.403 ^a^	67 (14)
**Gender**			0.985 ^b^	
Male (%)	20 (52.6 %)	18 (47.4 %)		38 (64.6 %)
Female (%)	11 (52.4 %)	10 (47.6 %)		21 (35.6 %)
**Race**			0.216 ^c^	
Malay (%)	21 (67.7 %)	13 (46.4 %)		34 (57.6 %)
Chinese (%)	6 (19.4 %)	9 (32.1 %)		15 (25.4 %)
Indian (%)	4 (12.9 %)	4 (14.3 %)		8 (13.6 %)
Others (%)	0 (0 %)	2 (7.2 %)		2 (3.4 %)
BMI (kg/m^2^)	28.1 ± 4.4	29.1 ± 5.0	0.403 ^d^	28.5 ± 4.7
SBP (mmHg)	134 ± 14	129 ± 12	0.149 ^d^	132 ± 13
DBP (mmHg)	76.7 ± 8.9	75.3 ± 9.7	0.557 ^d^	76 ± 9
Duration of diabetes (year)	15.3 ± 7.6	17.9 ± 8.9	0.231 ^d^	16.5 ± 8.3
HbA1c (%)	7.59 ± 0.95	7.56 ± 1.10	0.903 ^d^	7.57 ± 1.02
eGFR (mL/min/1.73 m^2^)	54.9 ± 18.4	56.0 ± 20.4	0.829 ^d^	55.9 ± 19.2
Serum creatinine (µmol/L)	122 ± 48	118 ± 34	0.829 ^d^	121 ± 42
UACR (mg/mmol)	20.9 (45.6)	18.8 (43.9)	0.988 ^a^	19 (44.3)
Urea (mmol/L)	6.95 ± 2.56	6.82 ± 2.07	0.832 ^d^	6.89 ± 2.32
Uric acid (mmol/L)	383 ± 102	427 ± 99	0.097 †^,d^	404 ± 13

All values are presented as mean ± standard deviation or median (IQR). ^a^ Mann-Whitney test. ^b^ Chi-square test. ^c^ Fisher’s Exact test. More than 20% of the cells have expected value of less than 5. ^d^ Independent *t*-test. † Log transformation was applied. HbA1c: glycated haemoglobin, UACR: Urine Albumin-Creatinine ratio, eGFR: estimated glomerular filtration rate, SBP: systolic blood pressure, DBP: diastolic blood pressure, BMI: Body Mass Index.

**Table 2:** Comparison between intervention and placebo group after six months of tocotrienol-rich vitamin E supplementation.

Parameters	2 Months Post-Supplementation				4 Months Post-Supplementation				6 Months Post-Supplementation			
	Intervention (31)	Placebo (28)	Mean Difference Mean (95%CI)	*p*-Value	Intervention (31)	Placebo (28)	Mean Difference Mean (95%CI)	*p*-Value	Intervention (31)	Placebo (28)	Mean Difference Mean (95%CI)	*p*-Value
**General**												
HbA1c (%)	7.74 ± 1.16	7.87 ± 1.36	−0.16 (−0.62, 0.29)	0.480 ^a^	-	-	-	-	7.43 ± 1.26	7.31 ± 1.06	0.082 (−0.47, 0.64)	0.769 ^a^
Systolic BP—mmHg	132 ± 15	129 ± 14	−1.95 (−9.87, 5.98)	0.624 ^a^	136 ± 15	130 ± 13	1.39 (−6.62, 9.41)	0.729 ^a^	136 ± 14	133 ± 13	−1.78 (−9.99, 6.43)	0.666 ^a^
Diastolic BP—mmHg	74.4 ± 8.90	75.0 ± 9.32	−2.01 (−6.09, 2.08)	0.329 ^a^	77.3 ± 9.30	72.9 ± 9.30	2.98 (−1.26, 7.24)	0.165 ^a^	77.8 ± 8.66	77.7 ± 9.26	−1.27 (−5.16, 2.61)	0.516 ^a^
**Renal Parameters**												
Serum creatinine—µmol/L	118 ± 42.1	127 ± 39.3	−13.4 (−22.1, −4.7)	0.003 ^a,^*	117 ± 43.4	122 ± 41.1	−10.4 (−19.9, −0.87)	0.033 *^,a^	117 ± 44	122 ± 38	−9.66 (−18.49, −0.83)	0.033 *^,a^
eGFR—mL/min/1.73 m^2^	56.3 ± 18.7	51.4 ± 19.3	6.02 (2.18, 9.87)	0.003 ^a,^*	57.2 ± 18.1	54.1 ± 20.8	4.2 (−0.01, 8.5)	0.051 ^a^	58.1 ± 18.9	53.4 ± 19.7	5.69 (−0.96, 10.43)	0.019 *^,a^
UACR—mg/mmol	13.8 (45)	13.4 (27)	-	0.264 ^b^	-	-	-	-	23.2 (46.7)	9.55 (49.9)		0.574 ^b^
Urea – mmol/L	7.69 ± 2.46	7.90 ± 2.48	−0.33 (−1.31, 0.64)	0.498 ^a^	7.43 ± 2.37	7.30 ± 2.10	−0.01 (−0.79, 0.78)	0.984 ^a^	7.26 ± 2.31	7.35 ± 2.65	−0.22 (−1.19, 0.74)	0.644 ^a^
Uric acid – mmol/L	358 ± 121	418 ± 89.6	−16.7 (46.4, 13.0)	0.265 ^a^	387 ± 113	414 ± 91	17.0 (−10.0, 44.3)	0.216 ^a^	395 ± 130	431 ± 105	7.89 (−28.6, 44.4)	0.667 ^a^
**Biomarkers**												
TGF-β1 – ng/mL	23.3 (19.0)	23.0 (11.4)	-	0.933 ^b^	-	-	-	-	21.72 (7.5)	17.4 (8.4)	-	0.534 ^b^
VEGF-A—pg/mL	862 ± 473	763 ± 472	-	0.127 ^a^	-	-	-	-	796 ± 488	551 ± 356	-	0.794 ^a^

Data are presented as mean ± standard deviation or Median (IQR). ^a^ Independent *t*-test. ^b^ Mann Whitney U test. * Significant at *p* < 0.05. HbA1c: Glycated haemoglobin, UACR: Urine Albumin-Creatinine Ratio, eGFR: Estimated Glomerular Filtration Rate, Systolic BP: Systolic blood pressure, Diastolic BP: Diastolic blood pressure, TGF-β1: Transforming growth factor beta 1, VEGF-A: Vascular endothelial growth factor A.

**Table 3:** Comparison between intervention and placebo group after twelve months of tocotrienol-rich vitamin E supplementation.

Parameters	8 Months Post-Supplementation				10 Months Post-Supplementation				12 Months Post-Supplementation			
	Intervention (31)	Placebo (28)	Mean Difference Mean (95%CI)	*p*-value	Intervention (31)	Placebo (28)	Mean Difference Mean (95%CI)	*p*-Value	Intervention (31)	Placebo (28)	Mean Difference Mean (95%CI)	*p*-Value
**General**												
HbA1c (%)	7.88 ± 1.44	7.97 ± 1.27	−0.13 (−0.78, 0.53)	0.698 ^a^	7.68 ± 1.36	8.86 ± 5.87	−1.02 (−3.26, 0.83)	0.239 ^a^	7.75 ± 1.41	7.98 ± 1.22	−0.26 (−0.32, 0.38)	0.424 ^a^
Systolic BP—mmHg	135 ± 13	129 ± 11	1.39 (−6.17, 8.94)	0.715 ^a^	132 ± 15	132 ± 12	−4.76 (−13.0, 3.48)	0.252 ^a^	135 ± 13	131 ± 10	−0.68 (3.79, −6.91)	0.858 ^a^
Diastolic BP—mmHg	74.2 ± 8.26	74.6 ± 10.5	−1.86 (−5.87, 2.14)	0.355 ^a^	72.9 ± 10.5	75.9 ± 10.9	−4.40 (−8.94, 0.15)	0.058 ^a^	74.2 ± 9.16	76.0 ± 10.4	2.01 (−7.22, 0.82)	0.117 ^a^
**Renal Parameters**												
Serum creatinine—µmol/L	118 ± 41.3	127 ± 45.0	−13.4 (−23.9, −2.95)	0.029 *^,^†	116 ± 41	120 ± 37	−8.75 (−18.1, 0.59)	0.066 ^a^	114 (38.8)	107 (40.2)	-	0.076 ^b^
eGFR—mL/min/1.73 m^2^	56.7 ± 18.4	52.6 ± 21.0	5.19 (1.22, 9.16)	0.011*	56.8 ± 16.1	54.2 ± 19.3	3.69 (−0.93, 8.32)	0.115 ^a^	56.8 ± 16.7	53.8 ± 20.2	4.07 (−0.522, 8.66)	0.081 ^a^
UACR—mg/mmol	-	-	-	-	-	-	-	-	28.6 (56.4)	15.3 (45.8)		0.125 ^b^
Urea – mmol/L	6.40 ± 2.55	7.54 ± 2.85	5.19 (1.22, 9.16)	0.014 *	6.83 ± 2.02	7.07 ± 1.90	−0.37 (−1.24, 0.49)	0.394 ^a^	6.68 (2.18)	7.43 (4.02)		0.021 *^,b^
Uric acid – mmol/L	401 ± 118	426 ± 101	19.2 (−18.7, 57.2)	0.314 ^a^	385 ± 110	399 ± 80.5	30.1 (−1.94, 62.3)	0.065 ^a^	372 ± 83	416 ± 106	0.049 (−37.8, 37.9)	0.998 ^a^
**Biomarkers**												
TGF-β1 – ng/mL	-	-	-	-	-	-	-	-	19.0 (8.7)	14.6 (7.3)	-	0.638 ^b^
VEGF-A pg/mL	-	-	-	-	-	-	-	-	653(429)	497 (511)	-	0.940 ^a^

Data are presented as mean ± standard deviation or Median (IQR). ^a^ Independent T-test. ^b^ Mann Whitney U test. * Significant at *p* < 0.05. † Log transformation was applied for data analysis, HbA1c: Glycated haemoglobin, UACR: Urine Albumin-Creatinine Ratio, eGFR: Estimated Glomerular Filtration Rate, Systolic BP: Systolic blood pressure, Diastolic BP: Diastolic blood pressure, TGF-β1: Transforming growth factor beta 1, VEGF-A: Vascular endothelial growth factor A.

**Table 4:** Correlation between renal parameters with TGF-β1 and VEGF-A.

Baseline Parameters	TGF-β1 – ng/mL		VEGF-A pg/mL	
	Correlation, *r*	*p*-Value ^a^	Correlation, *r*	*p*-Value ^a^
**Serum creatinine—µmol/L**	−0.036	0.785	−0.062 ^a^	0.638
**eGFR—mL/min/1.73 m^2^**	0.054	0.687	0.119 ^a^	0.370 *

^a^ Spearman’s correlation. Assumption fulfilled. * Correlation is significant at *p* < 0.05. TGF-β1: Transforming growth factor beta 1, VEGF A: vascular endothelial growth factor-A, eGFR: estimated glomerular filtration rate.

**Table 5:** Subgroup analysis comparing tocotrienol-rich vitamin E supplementation between intervention and placebo group at 6 months.

Parameters (Subgroup)	2 Months Post-Supplementation			4 Months Post-Supplementation			6 Months Post-Supplementation		
	Intervention (31)	Placebo (28)	*p*-Value	Intervention (31)	Placebo (28)	*p*-Value ^a^	Intervention (31)	Placebo (28)	*p*-Value
**General**									
HbA1c (%)	0.05 ± 1.08	0.44 ± 0.85	0.295 †^,a^	-	-	-	−0.090 ± 1.27	−0.34 ± 0.86	0.505 ^a^
Systolic BP—mmHg	0.05 ± 15.77	−0.82 ± 13.41	0.858 ^a^	2.13 ± 15.23	1.88 ± 14.88	0.962	4.56 ± 16.58	6.08 ± 15.82	0.778 ^a^
Diastolic BP—mmHg	−2.00 ± 9.84	−1.77 ± 6.17	0.933 ^a^	0.57 ± 8.35	−2.41 ± 5.15	0.210	1.54 ± 8.54	3.51 ± 7.12	0.455 ^a^
**Renal Parameters**									
Serum creatinine—µmol/L	−5.88 ± 18.66	12.45 ± 16.58	0.004 ^a,^*	−9.55 ± 8.92	−7.23 ± 17.68	0.046 ^a,^*^,^†	−7.23 ± 17.68	5.82 ± 21.38	0.050 ^a,*^
eGFR—mL/min/1.73 m^2^	1.65 ± 6.70	−4.18 ± 6.30	0.010 ^a,^*	4.36 ± 9.38	−3.41 ± 4.27	0.003 ^a,^*	4.55 ± 11.06	−1.11 ± 7.09	0.078 ^a^
UACR—mg/mmol	0.10 (8.83)	−0.70 (10.65)	0.177 ^b^	-	-	-	0.60 (27.08)	0.50(7.60)	0.497 ^b^
Urea – mmol/L	0.84 ± 2.06	0.68 ± 1.82	0.808 ^a^	0.45 ± 1.82	0.30 ± 1.27	0.777	−0.40 (1.03)	0.00(2.40)	0.729 ^b^
Uric acid – mmol/L	−17.9 ± 44.7	−11.8 ± 65.5	0.744 ^a^	−5.95 ± 43.9	−1.19 ± 40.0	0.734	17.5 ± 77.6	16.7± 72.1	0.973 ^a^

Data are presented as mean ± standard deviation or Median (IQR). ^a^ Independent T-test. ^b^ Mann Whitney U test. * Significant at *p* < 0.05. † Log transformation was applied for data analysis, HbA1c: Glycated haemoglobin, UACR: Urine Albumin-Creatinine Ratio, eGFR: Estimated Glomerular Filtration Rate, Systolic BP: Systolic blood pressure, Diastolic BP: Diastolic blood pressure.

**Table 6:** Subgroup analysis comparing Tocotrienol-rich vitamin E supplementation between intervention and placebo group at 12 months.

Parameters (Subgroup)	8 Months Post-Supplementation			10 Months Post-Supplementation			12 Months Post-Supplementation		
	Intervention (31)	Placebo (28)	*p*-Value	Intervention (31)	Placebo (28)	*p*-Value	Intervention (31)	Placebo (28)	*p*-Value
**General**									
HbA1c (%)	0.32 ± 1.38	0.30 ± 0.98	0.969 †^,a,^*	-	-	-	0.42 ± 1.65	0.49 ± 6.78	0.879 ^a^
Systolic BP—mmHg	1.77 ± 16.40	−0.88 ± 10.75	0.573 ^a^	1.57 ± 17.79	0.65 ± 10.89	0.854	3.79 ± 19.59	2.12 ± 8.23	0.746 ^a^
Diastolic BP—mmHg	−3.38 ± 6.64	−1.47 ± 9.84	0.489 ^a^	−3.58 ± 9.75	−1.71 ± 7.25	0.518	−2.45 ± 8.44	0.88 ± 7.48	0.558 ^a^
**Renal Parameters**									
Serum creatinine—µmol/L	−7.29 ± 14.31	13.8 ± 27.4	0.008 ^a,^*	−11.15 ± 17.66	1.91. ± 19.50	0.040 *	−7.85 (20.75)	0.84 (26.03)	0.042 ^b^
eGFR—mL/min/1.73 m^2^	2.35 ± 5.43	−3.29 ± 9.10	0.026 ^a,^*	5.09 ± 6.34	−0.13 ± 6.85	0.021 *	4.83 ± 6.78	−1.45 ± 9.18	0.022 ^a,^*
UACR—mg/mmol	-	-	-	-	-	-	1.78 (45.7)	0.56 (17.04)	0.141 ^b^
Urea – mmol/L	−0.72 ± 2.13	0.79 ± 1.59	0.021 ^a,^*	−0.58 ± 1.45	−0.17 ± 1.59	0.421	−0.87 ± 1.69	0.81 ± 2.39	0.017 ^a,^*
Uric acid – mmol/L	−12.0 (103)	6.00 (104)	0.916 ^b^	−12.36 ± 70.75	−25.71 ± 57.89	0.545	−22.07 ± 63.88	1.45 ± 82.73	0.336 ^a^

Data are presented as mean ± standard deviation or Median (IQR). ^a^ Independent T-test. ^b^ Mann Whitney U test. * Significant at *p* < 0.05. † Log transformation was applied for data analysis, HbA1c: Glycated haemoglobin, UACR: Urine Albumin-Creatinine Ratio, eGFR: Estimated Glomerular Filtration Rate, Systolic BP: Systolic blood pressure, Diastolic BP: Diastolic blood pressure.

**Table 7:** Renal parameters at 12 months Tocovid supplementation and at 6 months post washout between intervention and placebo groups.

Renal Parameters	Intervention Group (*n* = 29)			Placebo (*n* = 27)			*p*-Value
	At 12 Months	6-Months Washout	Change	At 12 Months	6-Months Washout	Change	
HbA1c (%)	7.50 ± 1.20	7.50 ± 1.30	−0.40 ± 1.00	8.00 ± 1.20	7.70 ± 1.20	−0.30 ± 0.80	0.426
UACR (mg/mmol)	28.60 (56.40)	15.80 (34.20)	−19.30 ± 25.70	15.30 (45.80)	18.50 (56.20)	4.60 ± 17.90	0.006 *
Serum creatinine (umol/L)	107.00 (37.10)	116.70 (42.40)	9.70 ± 1.60	102.6 (47.20)	108.70 (63.90)	6.10 ± 3.00	0.657
eGFR (ml/min/1.73 m^2^)	61.70 ± 18.20	50.90 ± 15.50	−10.80 ± 22.20	59.00 ± 21.40	54.70 ± 19.20	−4.30 ± 12.80	0.212
Urea (mmol/L)	6.20 ± 1.60	7.70 ± 2.50	1.50 ± 2.40	7.20 ± 2.30	7.20 ± 2.40	0.10 ± 2.20	0.026 *
Uric acid (mmol/L)	370.30 ± 87.60	355.90 ± 73.20	−5.60 ± 24.40	382.30 ± 78.70	392.30 ± 92.90	10.00 ± 63.20	0.250

Data are presented as mean ± standard deviation or median (interquartile range). * Significant at *p* < 0.05. *p* values obtained using independent *t*-test comparing changes in intervention and placebo group. UACR and serum creatinine presented as median (IQR). *p*-value obtained using Mann–Whitney test. eGFR, estimated glomerular filtration rate; HbA1c, glycated hemoglobin.

## Data Availability

Data presented in this study is available on request.
